# Disrupting Management Research? Critical Reflections on *British Journal of Management* COVID‐19 Research and an Agenda for the Future

**DOI:** 10.1111/1467-8551.12699

**Published:** 2022-12-16

**Authors:** Stephen Brammer, Layla Branicki, Martina Linnenluecke

**Affiliations:** ^1^ School of Management University of Bath Bath BA2 7AY UK; ^2^ Macquarie Business School Macquarie University Macquarie Park NSW 2113 Australia

## Abstract

Research interest in extreme contexts was growing before the COVID‐19 pandemic and has intensified since. The climate crisis, significant geo‐political conflict, political polarization and upheaval, and economic/financial crises that present existential challenges to organizations have all contributed to rising interest in extreme‐context research. COVID‐19 itself has generated an enormous body of research across all sub‐fields of management. However, the substantive, methodological and conceptual implications of this large volume of research remain unclear. In this introduction to the *British Journal of Management* COVID‐19 Online Virtual Issue, we describe and analyse COVID‐19 research so far published in the *British Journal of Management*. The Journal was proactive in seeing the profound implications of COVID‐19 for management research and practice, issuing an early call for contributions, and publishing several exploratory commentaries as early as July 2020. In this paper, we situate COVID‐19 research within the broader extreme‐context research, analyse contributions made so far, and build upon an extended taxonomy of extreme contexts to suggest ways for future research to generate further impactful insights.

## Introduction

The global COVID‐19 pandemic has generated a huge volume of management research. Over 8000 articles that focus on the COVID‐19 pandemic have been published so far in business and management, including 27 articles in the *British Journal of Management*. The first wave of this research prospectively examined the potential implications of the pandemic for particular sub‐fields of business and management research. For example, articles examined the impacts of COVID‐19 on international business (Branicki, Sullivan‐Taylor and Brammer, 2021; Verbeke and Yuan, 2021), tourism (Sigala, 2020; Zenker and Kock, 2020), marketing and consumer behaviour (Roggeveen and Sethuraman, 2020; Sheth, 2020), supply chain management (Sarkis, 2020), public management (Ansell, Sørensen and Torfing, 2021), and human resource management (Collings *et al.*, 2021; Hamouche, 2021). As the pandemic progressed, research started to increasingly focus on the lived experience of the pandemic (e.g., Branicki, Sullivan‐Taylor and Brammer, 2021; Plotnikof *et al.*, 2020; Pradies *et al.*, 2021) and began to identify the broader societal and economic relevance of the pandemic, for example for small businesses (e.g., Dyduch *et al.*, 2021; Klyver and Nielsen, 2021), the deepening of inequalities (e.g., Dang and Nguyen, 2020; Zheng and Walsham, 2021), and wellbeing relative to socio‐economic status (Wanberg *et al.*, 2020).

However, while business and management research concerned with COVID‐19 has proliferated, we so far know very little regarding the overall emphasis, character, and patterns within this research, and therefore know little regarding its likely longer‐term implications for future research and practice. Verma and Gustafsson (2020) conducted a bibliometric analysis of 107 articles published in journals indexed in Scopus and the Web of Science between January and May 2020. Using a co‐word occurrence analysis, they identified four broad clusters of management research that examined: (1) the overall impact of COVID‐19 on business; (2) COVID‐19 and technology; (3) COVID‐19 and supply chain management; and (4) COVID‐19 and the service industry. Similarly, Piccarozzi, Silvestri and Morganti (2021) examined a broader range of literature (studies published before 10 February 2021, totalling 159 papers in the Scopus database) and conducted a systematic descriptive analysis of those papers. Among other findings, these authors highlighted the broad balance between conceptual and empirical papers on COVID‐19, identified two new topic clusters (relative to Verma and Gustafsson) focusing on consumer behaviour and corporate social responsibility, and drew attention to the relatively diffuse nature of the literature.

Research on COVID‐19 has progressed alongside the evolving pattern of the pandemic, and now that more than 2 years have passed since the initial wave of the novel coronavirus, it is timely to reflect on the contributions of COVID‐related business and management research, particularly within extreme‐context research more broadly. Extreme contexts are those in which events occur that present existential threats to organizations operating within them (Hannah *et al.*, 2009), and have been the subject of growing attention in research since before the pandemic (see Hällgren, Rouleau and De Rond, 2018). At the same time, extreme‐context research has so far only “scratch[ed] the surface of a field in dire need of more empirical evidence” (Hällgren, Rouleau and De Rond, 2018, p. 112), and remains partial in the sense that “relative to advances in emergency and risky contexts, research on disrupted contexts is still in its infancy” (Hällgren, Rouleau and De Rond, 2018, p. 145). Therefore, in addition to the substantive interest in analysing the contributions of business and management scholars to evaluating the impacts of the pandemic, COVID‐19 presents a rare opportunity to significantly advance extreme‐context research.

This introduction to the *British Journal of Management* COVID‐19 Online Virtual Issue contributes to these debates by critically examining research concerned with COVID‐19 published in the *British Journal of Management*, and by situating this existing research within extreme‐context research. Our analysis sees research published in the *British Journal of Management* as a microcosm of trends in the broader field and seeks both to critically reflect on the contributions of pandemic‐related research and to suggest how the unique possibilities afforded by COVID‐19 might be leveraged in future research. In particular, we ask: (1) How has the nature, emphasis, and focus of management research concerned with COVID‐19 evolved throughout the pandemic, and (2) what gaps, issues, and potential for future management research flow from the current state of that research?

The main contribution of the paper is a framework that describes relevant focal phenomena, processes and relationships across three levels of analysis (Societal, Organizational, and Individual) and three temporal stages of the pandemic (Pre‐Pandemic, Within‐Pandemic, and Through/Post‐Pandemic), building on Hällgren, Rouleau and De Rond's (2018) existing taxonomy of extreme‐context research. While significant contributions have been published across these various levels and temporal dimensions, the rapidity with which research was undertaken coupled with the timing of research relative to the emerging pandemic suggest some limitations that future research could address. Particularly, we see a greater opportunity for the role of extreme contexts in theorizing and encourage researchers to pay greater attention to *recovery contexts* and *renewal contexts*, and suggest avenues for future research.

## Characterizing COVID‐19 as an extreme context

Recognition of the significance of extreme events for management, organization, and strategy has grown substantially in recent years. Even before COVID‐19, the salience of financial crises, impactful natural disasters, the growing climate crisis, and endemic political and social instability have encouraged the emergence of important new research (Hällgren, Rouleau and De Rond, 2018; Rouleau, Hällgren and de Rond, 2021; Wenzel, Stanske and Lieberman, 2020). Notwithstanding the research effort underway before the pandemic, COVID‐19 has undoubtedly both vindicated pre‐existing warnings by research(ers) about the possibility of a global pandemic and accelerated and broadened interest among management scholars regarding how organizations experience and navigate extreme events.

Given the proliferation of terminology regarding crises, disasters as well as “extreme” events and contexts (Hällgren, Rouleau and De Rond, 2018), it is useful to define our focus. Focusing on extreme events as they relate to the organizational level of analysis, the “extremeness” of an event can be judged relative to three necessary criteria, specifically that an event has: “(1) the potential to cause massive physical, psychological, or material consequences that occur in physical or psychosocial proximity to organization members, (2) the consequences of which are thought unbearable by those organization members, and (3) are such that they may exceed the organization's capacity to prevent those extreme events from actually taking place” (Hannah *et al.*, 2009, p. 898). Reflecting this, extreme contexts are those “where one or more extreme events are occurring or are likely to occur that may exceed the organization's capacity to prevent and result in an extensive and intolerable magnitude of physical, psychological, or material consequences to – or in close physical or psychosocial proximity to – organization members” (Hannah *et al.*, 2009, p. 898). The scale, scope and variety of impacts of COVID‐19 clearly qualify it as an extreme event, thus providing an extreme context for organizations (Rouleau, Hällgren and de Rond, 2021).

Extreme‐context research in business and management is extremely varied, though there are some larger clusters of related work within the overall body of research (Hällgren, Rouleau and De Rond, 2018). Studies that examine organizational experiences of extreme events often draw upon retrospective accounts and documentary evidence (see Linnenluecke, 2017). Much of the organization‐level research on extreme contexts focuses on unpacking the processes by which extreme events came about within what Hällgren, Rouleau and De Rond (2018) would call “risky” contexts (i.e., those activities in which there is an inherent risk of an adverse event's occurrence, such as space exploration, industrial processes or aviation). This research typically entails rich ex‐post analyses of significant events such as the Bhopal and Challenger space shuttle disasters, highlighting the faulty cognitive, decision‐making, and communication processes through which smaller issues escalated into more extreme events (see Hällgren, Rouleau and De Rond, 2018). Other related research examines organizational experiences with and responses to disruptions (i.e., shocks unrelated to an organization's core activities). Disruptive events are recognized as highly heterogeneous, and as especially difficult for organizations to respond to because of their unanticipated nature. As for research in emergency contexts, disrupted‐context research is typically retrospective in its approach, examining how mundane aspects of organizations (their structures, processes, routines, and hierarchies) tend to render them inflexible in the face of disruptions. Events such as hurricanes and earthquakes often provoke the creation of new, temporary, organizational forms in which pre‐existing organizations, civil society actors, government and other stakeholders collaborate to meet the specific challenges of disrupted contexts (e.g., Linnenluecke and McKnight, 2017; Shepherd and Williams, 2014).

Research also focuses on how organizations recover and learn from extreme events. Given the retrospective nature of much of the extreme‐context research, a considerable body of research examines when, and if, organizations learn from experiencing extreme contexts, often emphasizing the lack of learning that occurs (Lampel, Shamsie and Shapira, 2009; Starbuck, 2009). A large amount of research has examined how organizations – and other actors – retrospectively construct and communicate about a given extreme event to manage stakeholder relations and maintain legitimacy (see Hällgren, Rouleau and De Rond, 2018). Finally, studies emphasize individual‐level experiences of extreme contexts. These studies expose rich evidence regarding the role of individuals, often in their professional roles, in navigating extreme contexts. The research seeks to understand what factors enable individuals to cope with extreme contexts and to sustain themselves in “dirty work”, as well as showcasing the personal heroism and courage often seen when extreme events are encountered (e.g., Dick, 2005; Quinn and Worline, 2008).

To summarize, considerable prior extreme‐context research exists, with particular emphases on (1) the organizational and individual levels of analysis, (2) the within‐ and post‐event temporalities, and (3) qualitatively examining extreme events and contexts, typically through retrospective case analyses drawing on secondary sources.

## Findings

In this section, we reflect upon articles published in the *British Journal of Management* that address the issues, challenges, and experiences of the COVID‐19 pandemic. To date, 27 *British Journal of Management* articles have been published that examine aspects of the pandemic. We begin by offering a broad descriptive overview of this research, before conducting a detailed thematic analysis.

### Descriptive overview

Research prompted by the pandemic, like the pandemic itself, consists of several distinct phases. The first five articles published in the Journal were published in the July 2020 issue. Reflecting the nascency and proximity of the pandemic, these essays necessarily aimed to evaluate the likely impacts of the pandemic from, with the benefits of hindsight, a relatively early stage. Three of the essays (Beech and Anseel, 2020; Brammer and Clark, 2020; Budhwar and Cumming, 2020) reflected on potential impacts on higher education, and on business education and research in particular. The remaining two (Shankar, 2020; Verbeke, 2020) examined potential impacts in contexts (global supply chains, IT) that were subject to early disruption or salience during the pandemic.

The second phase of published research was kicked off by Sheng *et al.*’s (2021) methodology corner article that reflected on the potential for methodological innovation to spur novel research in light of the possibilities of big data and new analytic techniques. Sheng *et al.*’s (2021) encouragement of methodological innovation is reflected in the seven other articles published in the October 2021 issue. Notably, six out of the seven early substantive articles deployed quantitative methodologies. Data sources included social media data (Wang *et al.*, 2021), experimental and quasi‐experimental approaches (Wang *et al.*, 2021; Klebe, Felfe and Klug, 2021), surveys (Koch and Schermuly, 2021; Mertzanis, 2021), and secondary financial databases (Tosun, Eshraghi and Muradoglu, 2021; Uddin and Chowdhury, 2021). Several of these studies examined pre‐pandemic data to draw inferences about likely pandemic impacts. The lone qualitative study in the first batch of empirical work is Yen *et al.*’s (2021) study of the particular challenges and coping strategies of migrants living in the UK.

The third phase of published pandemic research comprises the 14 studies published since January 2022. These studies are much more varied in respect of their methods, as might be expected given the longer window of time available to collect and analyse pandemic‐specific data. Seven out of the 14 are wholly or primarily qualitative in approach (Adisa *et al.*, 2022; Ashman *et al.*, 2022; Branicki, Kalfa and Brammer, 2022; Nayani *et al.*, 2022; Puthusserry *et al.*, 2022; Sahasranamam and Soundararajan, 2022; Wulandhari *et al.*, 2022). The other seven studies use quantitative approaches including experiments or quasi‐experiments (Kakarika *et al.*, 2022; Papagiannidis *et al.*, 2022), secondary financial databases (Ataullah, Le and Wood, 2022; Ghobadian *et al.*, 2022; Li, Trinh and Elnahass, 2022), and survey data (Calabrese, Cowling and Liu, 2022; Liu, Shahab and Hoque, 2022).

### Thematic analysis

**Figure 1:**
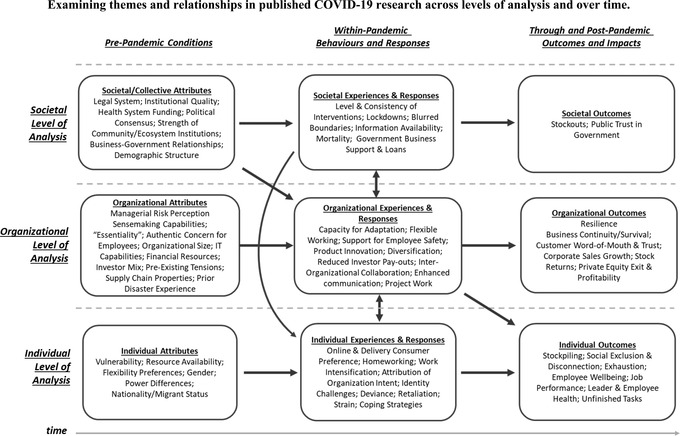
Examining themes and relationships in published COVID‐19 research across levels of analysis and over time

Overall, the research published in the *British Journal of Management* to date exhibits an impressive diversity of methods and approaches, given the urgency of undertaking the research. We turn now to a thematic analysis of the published studies, focusing our attention on the 21 substantive empirical studies. To structure our thematic analysis of the 21 studies published so far in the *British Journal of Management*, we draw upon Figure 1, below. Figure 1 distinguishes three levels of analysis (Societal, Organizational, and Individual) and three temporal stages of the pandemic (Pre‐Pandemic, Within‐Pandemic, and Through/Post‐Pandemic) to provide a framework to describe focal phenomena and processes (described within the nine ‘boxes’ of the framework), and relationships between them (described by the presence/absence of “arrows” between boxes). While recognizing the multiple interdependencies and relationships between levels of analysis and across time described in Figure 1, it is useful to adopt the level of analysis as a primary organizing schema for our thematic discussion.

#### Societal‐level analyses

The focus of four published studies lies at the societal/collective level, in recognition of the importance of institutional factors for outcomes at societal, and other, levels of analysis. Most clearly, Liu, Shahab and Hoque (2022) use global data from the International Coronavirus Survey of 178 countries between 20 March and 8 April 2020 to examine the influences on public trust in governments’ Covid‐response measures. Though situated early in the pandemic, Liu et al.’s study is relatively rare among the published work for the fact that their analysis encompasses all three phases of the pandemic (pre‐conditions, responses, outcomes). Their study shows that the provision of impartial, transparent and truthful government communications is vital for maintaining public trust, but also finds that the impacts of government responses on public trust vary significantly across countries’ legal systems. Additionally, Liu, Shahab and Hoque's (2022) analysis highlights the role of prior pandemic experience (especially of H1N1 and SARS) in bringing about more consensual public reactions to pandemic response strategies.

Calabrese, Cowling and Liu (2022) examine the take‐up of a key government economic intervention during the pandemic, namely financial support for affected businesses. Their analysis illuminates the scale and importance of two key financial support mechanisms for the overall financing of the SME sector during the early phases of the pandemic. For example, they show that over 90% of all debt funds provided to UK small‐ and medium‐sized enterprises in the early months of the pandemic were backed by the UK government, compared with less than 5% under normal circumstances.

Mertzanis (2021) draws upon data from the World Bank's large‐scale, multi‐phase, Enterprise Surveys of non‐financial firms in developing countries to examine the influence of countries’ epidemiological susceptibility risk (ESR) on firm performance (proxied by sales growth). A country's epidemiological susceptibility risk reflects its health infrastructure, environmental safety infrastructure, communications infrastructure, transport systems, demographic characteristics, economic activity, and institutional effectiveness. Mertzanis (2021) hypothesizes that country‐level institutions that are reflected in the ESR affect firms’ financial performance because they capture a country's capacity to identify, coordinate and respond to risks such as those seen during the pandemic. The study's evidence confirms the importance of ESR for firm performance both in pre‐pandemic conditions and during COVID‐19.

The last study with a predominant emphasis on a collective level of analysis (the ecosystem, rather than the society) is Sahasranamam and Soundararajan's (2022) study of innovation ecosystems that emerged in relation to two emergencies in India (the Kerala floods of 2018 and COVID‐19). Sahasranamam and Soundararajan (2022) study the emergence and enactment of ecosystem‐level agility that arises during emergencies and show that this agility rests on the strategic sensitivity, resource fluidity, and collective commitment demonstrated by actors in these emergencies. Moreover, their analysis highlights the formal and informal institutional arrangements that facilitate ecosystem‐level innovation in the face of an emergency.

Together, analyses at the societal level highlight the importance of pre‐pandemic conditions, especially in respect of institutional quality, for the nature and impact of pandemic‐response measures. Unsurprisingly, given the public salience of government responses, research emphasizes government actions and only addresses outcomes of interventions to a limited extent.

#### Organization‐level analyses

Eight published studies focus predominantly on the organization level of analysis, with five studies examining the nature of, and influences on, firms’ responses to COVID‐19, and three exploring the impacts of firm responses on organizational outcomes. Studies that examine how firms navigated the pandemic, including investigating the role of pre‐pandemic factors on such responses, highlight the wide range of phenomena that shaped how organizations managed the impacts of COVID‐19.

Several studies focus on the experiences of large, exchange‐listed, companies. For example, Ataullah, Le and Wood (2022) focus on the role of firms’ mix of investors in shaping how firms’ financial pay‐outs (dividends and share buybacks) were affected during COVID‐19. The authors hypothesize that the complex and nuanced needs and preferences of investors regarding the levels, form, and timing of anticipated financial returns materially affected whether firms maintained or cut payments to owners during the pandemic. Where a firm's ownership was composed more heavily of more patient owners seeking longer‐term capital growth, pay‐outs tended to be reduced, whereas where a firm's owners seek continuous income flows to meet obligations to their stakeholders (e.g., pension funds), cuts to pay‐outs are rarer.

Similarly, Ghobadian *et al.* (2022) explore the role of firms’ industry environment – in particular industry dynamism – in shaping the level of managerial attention to COVID‐19, contrasting US and Chinese firms. The authors hypothesize that dynamic industry environments present managers with more ambiguous and distracting conditions, reducing the level of attention paid to COVID‐19, finding strong support for this in their US, but not in their Chinese, sample of firms. Ghobadian *et al.* (2022) theorize that stronger business–government relations explain the insignificant effect among Chinese firms.

Other studies examining how organizations responded to COVID‐19 encompassed a broader range of organizational types. For example, Wulandhari *et al.* (2022) examine the role of managerial risk perceptions and organizational resilience among firms in the food sector, one area of the economy heavily affected by COVID‐19. One of the principal novelties of Wulandhari *et al.*’s study is the emphasis in their findings on the importance of pre‐existing organizational structures and modes of decision‐making for the processes of sensing emergent threats and developing responses to them.

Puthusserry *et al.* (2022) focus on the strategic adaptation of Indian digital SMEs through the pandemic, paying particular attention to the role of organizational designs and top management teams (TMTs) in firms’ approaches to crisis management. The authors’ findings include a typology of firms’ responses on two continuums, namely the structural centralization–decentralization and the homogeneous–heterogeneous nature of the TMTs’ social capital. The study also shows how different configurations of structure and capitals shape firms’ choices of mode and extent of adaptation.

Branicki, Kalfa and Brammer (2022) examine the role of human resource managers in helping organizations navigate COVID‐19. The authors introduce the concept of ‘societal paradox’ to describe the tension at the aggregate level between promoting health and prioritizing the economy, and explore how this tension manifests and is mitigated or exacerbated organizationally, depending on HR managers’ approach and the extent of pre‐existing organizational tensions. In addition to characterizing ‘societal paradoxes’, Branicki *et al.*’s (2022) contribution focuses on illuminating the role of middle managers as supporting actors in processes through which organizations navigate paradoxical tensions.

Like Branicki, Kalfa and Brammer (2022), Nayani *et al.* (2022) focus chiefly on the internal dynamics of how organizations dealt with some of the key challenges of COVID‐19 – in this case, the management of employee wellbeing. Nayani *et al.* (2022) propose that COVID‐19 provided organizations with an opportunity to authentically live values of mutuality in which employees and employing organizations recognize their shared interest in employee wellbeing. Their findings show the critical importance of attributions of authenticity to positive organization–employee relations, often grounded in concern for employee wellbeing that preceded the pandemic.

The second group of studies at the organization level focus on exploring the impacts of firm responses to COVID‐19 on firm‐level outcomes, again typically in the context of large, exchange‐listed companies. For example, Tosun *et al*. (2021) show that firms that survived the shock of a prior disaster – in this case, the 9/11 attacks – performed better, or less badly, than those that had no prior experience of disaster. Their analysis draws upon stock market reactions to COVID‐19, and suggests that prior disaster experience partly ‘immunized’ those firms with that experience to a different disaster two decades later. Relatedly, Li, Trinh and Elnahass (2022) examine the role of firms’ environmental and social (ES) activities on financial stability, in the context of a global sample of 244 commercial banks from 52 countries. Li, Trinh and Elnahass (2022) find that banks with higher levels of ES activities are more financially stable because they have stronger social capital, improved stakeholder relations, and less aggressive bank risk‐taking.

Overall, the organization level of analysis is covered by perhaps the richest and most diverse cluster of studies of COVID‐19 research, with these studies revealing numerous antecedents of organizations’ pandemic responses and experiences. To a significant extent, these confirm prior observations in the resilience literature (e.g., Linnenleucke, 2017) of the critical roles that financial and other resources and capabilities play in organizational experiences of shocks and crises. As with the societal level of analysis, the emphasis in existing research lies with the experience of, and response to, the pandemic, rather than with organizational impacts. Where impacts of pandemic responses are analysed, the emphasis is largely financial.

#### Individual‐level analyses

Studies that examine individual experiences of, and responses to, COVID‐19 make up the largest cluster of studies published so far in the Journal. The nine studies that focus principally on the individual level of analysis encompass a range of individual stakeholder roles, including customers/consumers (Papagiannidis *et al.*, 2022), employees/workers (Adisa *et al.*, 2022; Ashman *et al.*, 2022), and investors (Uddin and Chowdhury, 2021).

The impacts of the pandemic on consumer behaviour constitute one of the more memorable aspects of the early pandemic. Building on this, Papagiannidis *et al.* (2022) explore the nature and drivers of consumer stock‐piling behaviour and changing attitudes to online shopping. The authors used a pre‐study analysis of social media discourse regarding consumer behaviour during lockdown to identify concepts and issues for further study. Building on this, their online survey evidence showed that stockpiling was driven largely by emotional responses to uncertainty and that it helped increase consumer well‐being by reducing consumer anxiety and fear. Wang *et al.* (2021) explore the role of corporate responses to service failures on electronic word of mouth and trust recovery in the context of UK food retailers. The authors’ analysis examines social media responses to firms’ COVID‐19 announcements, finding that defensively framed messaging with emotional content tended to lead to more positive word of mouth. In a second quasi‐experimental analysis, Wang *et al.* (2021) found that emotionally framed corporate responses are associated with stronger consumer trust recovery.

Given the substantial impacts of the pandemic on work and workplaces, it is perhaps unsurprising that the majority of research at the individual level of analysis examined the experiences and responses of individuals in relation to their work and employment. Within this research, the changing and challenging nature of boundaries between work and home was a prominent theme. For example, Adisa *et al*. (2022) describe the lived experience of academics throughout the pandemic, highlighting employees’ paradoxically inflexible experience of flexible and home working. Additionally, these authors draw attention to the impacts of the pandemic on work intensification, increased surveillance by employers, and reduced social connection and support. Similarly, Ashman *et al.* (2022) explore the impacts of the pandemic on relationships between work and family. Uniquely among studies published so far in *British Journal of Management*, the gendered experience and impacts of COVID‐19 are foregrounded through a focus on employed mothers. Ashman *et al.*’s (2022) analysis illuminates how working mothers navigated the pandemic through processes of ‘re‐ordering’ that entailed maintaining self‐presentations as ‘ideal workers’, guilt regarding their incapacity to navigate the expectations of multiple roles, but also the enjoyment of the relative flexibility of home working. Koch and Schermuly (2021) emphasize the role of COVID‐19 in provoking emotional exhaustion among employees. They theorize that COVID‐19 generates exhaustion both directly and indirectly because it affects employees' capacity to complete tasks as planned. They further theorize that project management capabilities support employees in managing tasks, thereby reducing impacts on exhaustion. Drawing on both German and US samples and pre–post survey designs, they find strong support for their hypothesized relationships.

COVID‐19 has provoked significant discussion of leadership at multiple levels of society. It is unsurprising therefore that two studies focus explicitly on aspects of leadership during the pandemic. Kakarika *et al.* (2021) focus on the role of COVID‐19 as a contextual influence on supervisors’ propensity to retaliate against subordinates’ deviance. Specifically, the authors theorize that the pandemic entails a heightened emotional climate which simultaneously makes supervisors more sensitive to deviance and more empathetic towards subordinates experiencing the challenges of COVID‐19. In several quasi‐experimental studies and a field study, they found strong evidence that supervisor identity threat was associated with supervisor retaliation that strengthened throughout the pandemic, but was negatively moderated by the degree of COVID‐19‐induced empathic concern for co‐workers. Klebe, Felfe and Klug (2021) explore the effect of health‐related leadership (leadership emphasizing concern, value, and support for employee health) on employees throughout the pandemic. Specifically, they hypothesize the impacts of health‐related leadership on employee strain and performance and find that care for staff leads to reduced irritation among employees, additional employee effort, and lower employee exhaustion.

COVID‐19, like prior extreme events, affected individuals and groups in heterogeneous ways. Building on this observation, Yen *et al.* (2021) highlight the impacts of the pandemic on international migrants living in the UK, exploring how migrants from China, Italy and Iran developed coping strategies. Their findings highlight the hostile context experienced by migrants in the UK throughout the pandemic, as well as the paradox of migrants’ striving to cope with the hostile reactions provided by their initial coping strategies.

COVID‐19 has had profound and long‐lasting financial impacts on the economy. Uddin and Chowdhury (2021) provide a distinctive analysis of the impacts of the pandemic on one key financial actor – private equity investors. Generally, private equity investors are keen to sell their investments for a profit, having nurtured and developed the firms in which they invest. However, the pandemic contributed to depressed conditions within which the assets held by private equity investors might sell, calling for shifts in behaviour. Uddin and Chowdhury (2021) analyse 20 years of private equity fund data from across 79 countries. They find that COVID‐19 negatively affected deal values across all forms of private equity exit strategies. As a result, private equity investors tended to wait for a good time to exit rather than rushing to exit during the pandemic.

Research at the individual level of analysis encompasses individuals in various stakeholder roles and exposes the profoundly challenging nature of the pandemic for many people. In particular, the blurring of roles and the erosion of pre‐pandemic spheres are especially evident in published studies. Additionally, individual‐level research has the strongest emphasis on outcomes among existing research, highlighting impacts on well‐being and health as well as on work‐related performance.

## Discussion and conclusion

Having analysed COVID‐19‐related research published in the Journal so far, we now turn to reflecting on the contributions of this research and proposing an agenda for future research. The 27 papers published so far – notably all in print within 18 months of the start of the pandemic, and many much earlier than that – demonstrate a capacity for agility and responsiveness among researchers, reviewers, and the Journal itself that is both surprising and welcome in the context of longstanding critiques of the management publishing, and of associated limitations upon relevance and impact (Thorpe *et al.*, 2011). In that sense, research published during COVID‐19 testifies to the resilience and capacities of the management research community. Furthermore, the variety of research published, especially in methodological terms, is very encouraging and is notably more varied than prior extreme‐context research. The balance between quantitative and qualitative approaches, the use of quasi‐experimental methods, large‐scale samples and case study evidence all speak to a vibrant and innovative social science community.

While significant contributions have been published so far, the rapidity with which research was undertaken coupled with the timing of research relative to the emerging pandemic suggests some limitations that future research could address. First, existing published research has a relatively strong emphasis on the experience of the pandemic and a relatively limited focus on its implications, especially in the longer term. In that sense, extant research is perhaps rather descriptive, albeit in ways that showcase experiences and phenomena that have attracted limited attention within prior extreme‐context research. Second, existing research has – for understandable reasons – tended to emphasize particular organizational types and contexts over others, limiting the overall generalizability of the evidence presented. For example, much of the empirical research published so far has tended to focus on large corporate organizations or convenience samples of individuals. There is a lack of comprehensiveness to organizational coverage, and also a lack of explicitness regarding the role of sampling decisions and why particular samples generate important findings. Third, while published research has illuminated the pandemic in empirical terms, the broader and longer‐term implications of this research for novel concepts and theorization in management studies are, so far, rather limited. In that sense, the broader implications of COVID‐19 for management theory remain largely unexplored, something we reflect on and address in our next sub‐section.

### Future research: An extended taxonomy of extreme contexts

**Figure 2:**
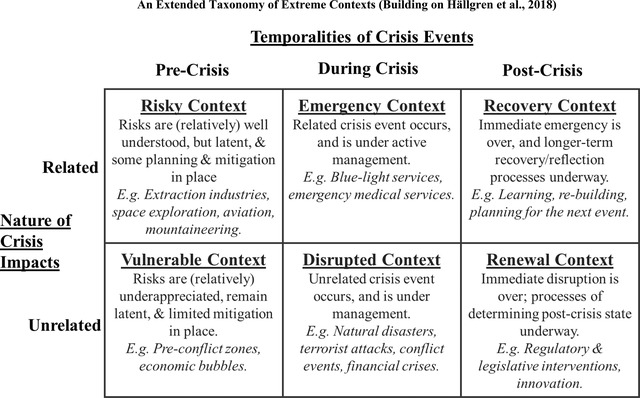
An extended taxonomy of extreme contexts (building on Hällgren, Rouleau and De Rond, 2018)

We propose that maximizing the value of future COVID‐related research, especially that with the potential to make more significant conceptual contributions, requires greater attention to the role of extreme contexts in theorizing. To facilitate this endeavour, we build upon and extend Hällgren, Rouleau and De Rond's (2018) taxonomy of extreme contexts. These authors distinguish between two characteristics of extreme contexts (whether events are potential or actual, and related or unrelated to an organization's core activities) to conceptualize four distinct extreme contexts. In Figure 2, we extend Hällgren, Rouleau and De Rond's (2018) framework in two ways. First, we extend the temporal scope over which extreme contexts are conceptualized to encompass three, rather than two, temporalities. Specifically, our extended framework sees event ‘potentiality’ as relating to the pre‐event or latent‐event temporality of an extreme context, ‘actual’ temporality as referring to the within‐event temporality within which an extreme event is being navigated or managed, and introduces a new ‘post‐event’ temporality to capture post‐event activities and processes. Second, because our objectives are not confined to analysing the state of existing extreme‐context literature, it is useful conceptually to characterize contexts that face the potential of unrelated extreme events. We call these contexts *Vulnerable contexts*.

Our framework encompasses the three contexts identified by Hällgren, Rouleau and De Rond (2018) as being the most common in prior research. Following Hällgren, Rouleau and De Rond (2018), we describe *Risky contexts* as contexts where an organization's core activity entails facing, near constantly, the relatively clearly understood risks of a particular extreme event's occurrence. Risky contexts have long been the study of the High‐Reliability Organizing (HRO) literature (e.g., Roberts, 1990; Weick, Sutcliffe and Obstfeld, 1999; Weick and Roberts, 1993). Hällgren, Rouleau and De Rond (2018) characterize two situations in which organizations encounter the experience of an extreme event. First, *Emergency contexts* describe circumstances where the extreme event is related to an organization's core activity, for example, accidents in the setting of aviation or space exploration, or extreme events in the context of the emergency services. Second, *Disrupted contexts* are those in which the extreme event is unrelated to a focal organization. For example, disrupted contexts are often those in which natural disasters or other events exogenous to organizations occur.

One of the notable features of the COVID‐19 pandemic from the standpoint of management research is that a single extreme event provides opportunities to examine organizations that will experience the pandemic as an emergency (e.g., those directly involved in promoting public health), and those that experience the pandemic as a disruption (e.g., organizations prevented from operating during lockdowns). Relatedness is a critical element of the organizational experience of extreme events because it shapes the extent to which organizations have developed experience, plans, specific resourcing and mitigation measures concerning extreme events. In light of this, we conceptualize *Vulnerable contexts* as those in which organizations have not experienced, and are not anticipating, an extreme event unrelated to their core activities, and are, as a result, unknowingly susceptible to such events occurring.

Considerable attention within prior extreme‐context research has been paid to the period following the immediate navigation of extreme events (Hällgren, Rouleau and De Rond, 2018). For this reason, our framework explicitly incorporates the post‐extreme event temporality. We describe *Recovery contexts* as those following an extreme event that is related to an organization's core activity. For example, research has examined how organizations learn from extreme events and build capacities to respond more effectively to future extreme events (e.g., Haunschild, Polidoro, and Chandler, 2015; Madsen, 2013). Importantly, recovery connotes striving to regain the pre‐extreme‐event state and to better navigate similar (related) extreme events in the future. We contrast *Recovery contexts* with *Renewal contexts*, where the latter describes contexts in which organizations experience the aftermath of an unrelated extreme event. Renewal differs from recovery in that while recovery typically encompasses a relatively clearly defined end state, renewal contexts are more ambiguous regarding desirable post‐crisis states.

Armed with our extended taxonomy, we can reflect on research generated so far during the pandemic. An initial observation is that almost all COVID‐19 research concerns disrupted (i.e., unrelated) rather than emergency (i.e., related) extreme contexts – something that stands in direct contrast to the prior literature. Emergency contexts are, of course, especially challenging to research directly, especially when the particular emergency is a public health crisis that presents considerable risk both to researchers and to those being researched. Nonetheless, the absence of emergency contexts and comparisons between emergency and disrupted contexts is an opportunity for important theoretical contributions regarding the capabilities, resources, behaviours, and approaches needed to navigate extreme contexts successfully. Relatedly, the pandemic generated new notions of ‘essentiality’ in relation to work/occupations, industry sectors, organizational functions and public service that have yet to materialize in management research.

A second observation is that relatively little research has so far examined the outcomes and impacts of pandemic navigation strategies and approaches, and where it has, the outcomes have largely been financial. The scope and diversity of experience of the pandemic have not yet been fully reflected in the research that has so far been published, and further analysis of individual, organizational, and societal heterogeneity regarding outcomes is warranted. The gendered nature of the pandemic experience has been noted elsewhere but has not been as prominently examined in mainstream management research as it should be.

A third opportunity for impactful future research comes from the greater emphasis on the role of temporality in extreme‐event experience, response, and outcomes. COVID‐19 is a distinctively long‐lived extreme event when compared with those examined in prior research. It has also varied in the nature and intensity of its ‘extremeness’ over time. Various aspects of temporality suggest themselves. The distinction between recovery (re‐establishing the pre‐pandemic state) and renewal (establishing a potentially distinct post‐pandemic state) and investigation of the antecedents of each at multiple levels of analysis hold the potential for very fruitful new research. Will the pandemic lead to any substantive long‐term change in how society, organizations, and individuals conceive of, prepare for, and experience future extreme events? Or, as for prior events, will the challenges and lessons of COVID‐19 soon be forgotten? How do processes of institutional change that might underpin long‐term change occur, and how might countervailing interests prevent that change? An examination of pre‐event practices, planning, and preparedness is almost absent from prior research, suggesting a significant research gap.

COVID‐19 has spurred a rapidly growing, methodologically and substantively diverse, and rapidly accessible body of management research. In contrast to much of the prior extreme‐context literature, which has often relied on retrospective accounts and secondary data, researchers drew on a wide range of methods and rapidly collected evidence of the experience of the pandemic. Perhaps for more than any other extreme event, management research has highlighted the day‐to‐day activities, practices, and behaviours provoked by the pandemic. However, the rich empirical insights afforded by this work have not yet delivered significant theoretical or conceptual developments, and neither has research sufficiently examined the impacts and outcomes of approaches to navigating the pandemic. Nonetheless, COVID‐19 has presented, and continues to present, significant opportunities to undertake management research of real relevance and societal impact, as well as the capacity to advance theory and practice regarding the extreme events that are an increasingly salient part of organizational life.
